# High frequency of colonization by extended-spectrum beta-lactamase-producing Gram-negative bacilli in hemodialysis patients and their household contacts in Colombia: dissemination between the community and the hospital

**DOI:** 10.4178/epih.e2022069

**Published:** 2022-08-27

**Authors:** Daniela Montoya-Urrego, Sara Tellez-Carrasquilla, Johanna M. Vanegas, Judy Natalia Jiménez Quiceno

**Affiliations:** 1 Grupo de Investigación en Microbiología Básica y Aplicada (MICROBA), Escuela de Microbiología, Universidad de Antioquia, Medellín, Colombia; 2 Grupo de Investigación en Salud Pública, Escuela de Ciencias de la Salud, Universidad Pontificia Bolivariana, Medellín, Colombia

**Keywords:** Colonization, Beta-lactam resistance, Gram-negative bacteria, Hemodialysis, Household contacts, Genetic variation

## Abstract

**OBJECTIVES:**

Increasing colonization by beta-lactam-resistant Gram-negative bacilli (BR-GNB) represents a risk for infections and bacterial resistance spread, both in hospitals and the community. Hemodialysis patients and their household contacts regularly transit between these environments. This study investigated the clinical and epidemiological characteristics of BR-GNB colonization in hemodialysis patients and their household contacts, as well as the genetic relationship between their isolates.

**METHODS:**

A cross-sectional study was conducted on hemodialysis patients at a hospital-associated dialysis center in Medellín, Colombia and their household contacts. Clinical and epidemiological information was collected. Colonization was assessed from stool or rectal swab samples. Bacterial identification and susceptibility were determined using chromogenic media and Vitek-2. Molecular characterization included beta-lactamase detection by polymerase chain reaction, multiple-locus sequence typing (MLST), pulsed-field gel electrophoresis, and identification of Escherichia coli phylogroups by the Clermont protocol.

**RESULTS:**

This study included 36 hemodialysis patients and 90 household contacts. Colonization by BR-GNB occurred in 58.3% of patients and 22.2% of household contacts. The main beta-lactamase detected was CTX-M group-1 (40.5%). In 3 of the 9 homes that had more than 1 colonized individual, a genetic relationship was found. MLST showed a high diversity in E. coli isolates, and the most frequent phylogroups were B1 and B2.

**CONCLUSIONS:**

These results show a high frequency of colonization and the presence of potentially pathogenic BR-GBN both in hospitals and the community. This highlights the importance of populations who move between those 2 environments, and the need to prevent the spread of bacterial resistance outside hospitals.

## INTRODUCTION

Beta-lactam-resistant Gram-negative bacilli (BR-GNB) have emerged as important pathogens that can cause a variety of healthcare-associated or community-acquired infections, and have been on the rise, according to the latest Centers for Disease Control and Prevention (CDC) reports [1]. Infections by this type of bacteria have serious implications in terms of increased mortality, longer hospital stays due to a delay in adequate treatment, and extra costs for health systems [1-3].

Hemodialysis patients present specific clinical characteristics that favor high frequencies of colonization by BR-GNB, even higher than those reported for other bacteria of clinical importance such as *Staphylococcus aureus*, therefore increasing the risk of infection [4]. In addition, these patients constantly circulate between hospital and community environments, so they act as carriers of these bacteria for long periods, facilitating their transmission to people with whom they interact closely, such as members of their family and their contacts within the community [5,6]. Likewise, hemodialysis patients share a link of care with their household contacts since they need constant accompaniment and assistance. Therefore, their household contacts also interact with the hospital environment and share spaces, objects, and habits with patients at home, which can facilitate the transmission of bacteria [7]. At the same time, different social, economic, epidemiological, cultural, and environmental factors converge in this population, which can favor colonization by resistant bacteria from community sources.

Accordingly, hemodialysis patients and their household contacts are a model population to understand the behavior of the colonization of BR-GNB between the hospital environment and the community. Taking into account that Colombia is an endemic country for BR-GNB, and that, particularly in Medellín, colonization by these microorganisms in hemodialysis patients has been reported in up to 52.7% [4], this study aimed to determine the clinical and epidemiological characteristics of colonization by BR-GNB in hemodialysis patients and their household contacts, in order to reach an understanding of the behavior of colonization between the hospital and the community, and to identify prevention and control strategies to reduce the spread of resistant bacteria.

## MATERIALS AND METHODS

### Study population

A cross-sectional study was conducted among hemodialysis patients and their household contacts between June 2019 and May 2020. The patients were treated at a dialysis center associated with a tertiary hospital in Medellín, which cares for approximately 350 patients. As selection criteria, hemodialysis patients had to share a home with at least 1 person; household contacts were defined as individuals who lived in the same house as the patient for a period equal to or greater than 6 months. For minors, consent was provided by their parents or legal guardians. Finally, if pets, such as cats and dogs, lived in the homes, they were screened.

Based on a total of 106 eligible hemodialysis patients, 70 were excluded for the following reasons: (1) 3 lived in temporary shelters or convents, (2) 6 lived alone, (3) 23 could not be contacted, (4) 29 refused to participate, and (5) 9 felt uncomfortable with sample collection. Meanwhile, of 146 eligible household contacts, 56 were excluded, since (1) 23 refused to participate and (2) 33 felt uncomfortable with the sample collection. A total of 126 participants distributed among 38 homes were included: 36 hemodialysis patients and 90 of their household contacts (Supplementary Material 1).

### Data collection

Hemodialysis patients were contacted by telephone from a cohort of a previous study in our research group, who presented previous colonization [4]. A visit to each home was arranged and, once informed consent had been provided, the clinical and epidemiological information was obtained using a form designed for this purpose. The information included general characteristics, clinical information, self-reports of hand-washing and eating habits (times per week). If there were pets in the home, the pets’ information and clinical history were included.

### Screening for beta-lactam-resistant Gram-negative bacilli colonization

Colonization by BR-GNB was evaluated based on stool samples of the patients and their household contacts, and rectal swabs from pets. Each sample was enriched in trypticase soy broth and incubated for 12 hours to 18 hours at 37°C. Subsequently, 100 µL of broth was seeded in the chromogenic media ChromID-ESBL and ChromID-CARBA (bioMérieux, Marcy-l’Étoile, France) for the selection of extended-spectrum beta-lactamase (ESBL)-producing and carbapenem-resistant Gram-negative bacilli [8,9]. To increase the sensitivity in the detection of carbapenem-resistant Gram-negative bacilli, the protocol validated by the CDC was also used, where the sample was enriched by adding an ertapenem disk (10 µg) and seeded on McConkey agar [10]. Bacterial identification and susceptibility testing were performed using the automated Vitek-2 system (bioMérieux). The antibiotics evaluated were amikacin, ampicillin/sulbactam, cefepime, cefoxitin, ceftazidime, ceftriaxone, ciprofloxacin, doripenem, ertapenem, gentamicin, imipenem, meropenem, piperacillin/tazobactam, and tigecycline, according to the Clinical and Laboratory Standards Institute cut-off points [11].

### Molecular detection of resistance mechanisms to beta-lactam antibiotics

DNA of the isolates was extracted using the Wizard Genomic DNA purification kit (Promega, Madison, WI, USA) according to the manufacturer’s instructions. Detection of beta-lactamases was carried out using polymerase chain reaction (PCR) amplification of genes that encode for CTX-M-G1, CTX-M-G2, CTX-M-G9, CTX-M-G8/25, TEM, and SHV [12]. Additionally, in carbapenem-resistant Gram-negative bacilli, the presence of genes encoding carbapenemase types KPC, IMP, VIM, NDM, and OXA-48 was evaluated by PCR, according to the protocols previously described and standardized in the laboratory [13,14].

### Molecular typing of isolates

In the event that at least 2 household members were colonized in the same home, the genetic relatedness of isolates from hemodialysis patients, household contacts, and pets was determined using pulsed-field gel electrophoresis (PFGE), which was performed using the XbaI restriction enzyme (Thermo Scientific, Waltham, MA, USA) [15]. The cluster analysis was performed using BioNumerics software version 6.0, and dendrograms were generated by the unweighted pair group method using average linkages. A similarity cutoff of 80% was used to define genetically related strains.

Additionally, multiple-locus sequence typing (MLST) was performed on a representative sample of the isolates, according to the group generated by PFGE [16]. The sequence type and the clonal complex were assigned using the web database (https://pubmlst.org/). Finally, all *Escherichia coli* isolates were typified using the Clermont method to establish the phylogroups that represented the highest clinical risk in colonized people. For this, quadruplex PCR and 2 simple PCRs were performed to recognize the 7 defined phylogroups (A, B1, B2, C, D, E, and F) according to the previously described protocol [17].

### Statistical analysis

Categorical variables were described with absolute and relative frequencies. Continuous variables were expressed with the mean and standard deviation or median and interquartile range (IQR), according to the assumption of normality. To determine the factors potentially associated with colonization in hemodialysis patients, a multivariate analysis using a generalized linear model for a Poisson distribution with a log-link function and a robust estimator of variance was performed. For household contacts, as there may be several per home, it was necessary to adjust for the cluster effect, so a generalized estimating equation model was performed for a Poisson distribution with a log-link function, assuming an exchangeable correlation and a robust estimator of variance. Each exposure was analyzed in a different model, adjusted for age; the association measures were prevalence ratios (PRs), expressed with their corresponding confidence interval (95% CI) and p-value. The statistical analysis was performed using Stata version 14.0 (StataCorp., College Station, TX, USA).

### Ethics statement

This study was approved by the Committee for Bioethics in Humans of the University of Antioquia (CBEIH-SIU), approval act 18-35-820, and by the Ethics Committee for Experimentation with Animals of the University of Antioquia (CEEA) with the minutes of session No. 136. All participants provided in formed consent.

## RESULTS

Most of the patients were men (55.6%, n= 20), the median age was 61 years old (IQR, 47-71), and the median number of household contacts per patient was 3 (IQR, 2-4). The main comorbidities were arterial hypertension (80.6%, n= 29) and diabetes mellitus (33.3%, n= 12). The clinical history revealed that 50% of patients (n= 18) had been hospitalized or undergone surgery in the last year, and 50% (n= 18) consumed antibiotics in the same period. Furthermore, 16.7% (n= 6) of patients had traveled in the last year, mainly locally.

Regarding the household contacts, the majority were women (71.1%, n= 64), and their median age was 39 years (IQR, 17-59). Their clinical histories revealed that 13.3% (n= 12) of the household contacts were hospitalized in the last year, and 30% (n= 27) consumed antibiotics in the same period. The main comorbidities reported were arterial hypertension (16.7%, n= 15) and diabetes mellitus (12.2%, n= 11). In addition, 31.1% of household contacts (n= 28) traveled in the last year. Other clinical and epidemiological data are summarized in Table 1.

### Colonization by beta-lactam-resistant Gram-negative bacilli

In general, colonization by BR-GNB was found in 32.5% (n= 41) of the participants. In hemodialysis patients, it was present in 58.3% of the patients (n= 21/36); 22 isolates were obtained, most of which were *E. coli* (77.3%, n= 17), followed by *Klebsiella pneumoniae* (13.6%, n= 3), *Klebsiella oxytoca* and *Pseudomonas aeruginosa* (4.5%, n= 1). Of the 22 isolates, 19 ESBL carriers were detected (86.4%), 2 were resistant to carbapenems, and 1 isolate was resistant to third-generation cephalosporins without the presence of ESBL but with a TEM-type beta-lactamase.

Among the household contacts, colonization was found in 22.2% (n= 20/90); 20 isolates were obtained, most of them *E. coli* (95%, n= 19) and 1 isolate of *P. aeruginosa*. Of the 20 isolates, 16 ESBL carriers were detected (80%), 1 was resistant to carbapenems, and 3 to third-generation cephalosporins without the presence of ESBL, but with a TEM-type beta-lactamase.

**Figure 1.**
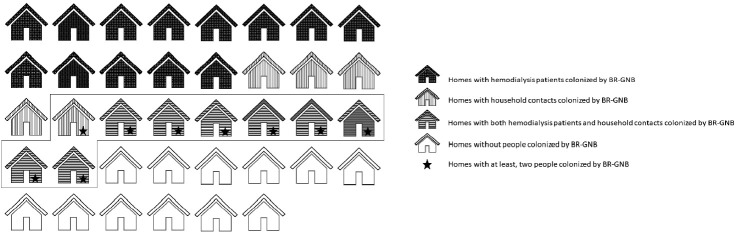
Colonization by beta-lactam-resistant Gram-negative bacilli (BR-GNB) in homes. Isolates from homes marked with a box were included in the pulsed-field gel electrophoresis analysis.

Of 38 homes evaluated, colonized people were found in 26 (68.4%), of which 13 homes had only colonized patients (50.0%), in 19.2% (n= 5) only colonized household contacts were found, and in 30.7% of homes (n= 8), colonized patients and household contacts were found (Figure 1). Colonization by *E. coli* of 2 or more members of the same home was detected in 23.6% (n= 9) of homes. Additionally, of the 25 pets evaluated (10 canines and 15 felines), colonization by BR-GNB was found in 2 dogs; both isolates were *E. coli*.

### Mechanisms of resistance to beta-lactams

**Figure 2.**
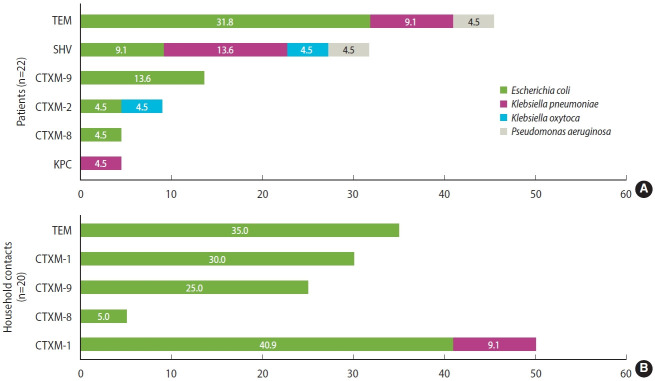
Mechanisms of resistance to beta-lactam-resistant Gram-negative bacilli (BR-GNB) isolated from hemodialysis (A) patients and (B) their household contacts.

Regarding resistance mechanisms, 83.3% (n= 35/42) of all isolates carried some type of ESBL. In hemodialysis patients, 50% of isolates carried ESBL CTX-M group 1, which includes CTX-M-1, CTX-M-3, and CTX-M-15 variants (40.9% *E. coli* and 9.1% *K. pneumoniae*), followed by CTX-M-9 with 13.6% (n= 3, *E. coli*); TEM and SHV beta-lactamases were also identified in a significant proportion of isolates (45.4% and 31.7%, respectively). Furthermore, 50% of the isolates carried at least 2 beta-lactamases simultaneously. In household contacts, the most common beta-lactamase was TEM (35%, n= 7), followed by ESBL CTX-M-group 1 (30%, n= 6), both detected in isolates of *E. coli*. Similarly, 20% of the isolates carried 2 beta-lactamases simultaneously. Regarding carbapenemases, KPC could be identified from a *K. pneumoniae* isolate. Importantly, NDM, OXA-48, VIM, or IMP type carbapenemases were not detected. All detected beta-lactamases are shown in Figure 2.

### Molecular typing of isolates

**Figure 3.**
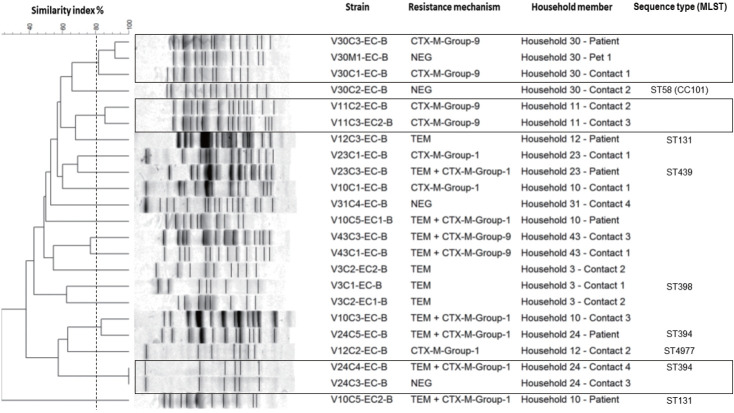
Genetic relationship between Escherichia coli isolates colonizing hemodialysis patients and their household contacts. The dotted line represents the cut-off point (80%) used to define the PFGE-related clones. The homes where there was a genetic relationship between the isolates are indicated in the box. PFGE, pulsed-field gel electrophoresis; MLST, multiple-locus sequence typing.

The genetic relatedness was evaluated in 9 homes, in which 2 or more inhabitants were colonized by *E. coli* (Figure 1). In total, 23 isolates were evaluated by PFGE, resulting in a genetic relationship between isolates from different members in 3 homes (33.3%); however, extensive diversity was identified in the *E. coli* isolates (Figure 3). It should be noted that in 1 family, there was a genetic relationship between the patient, 1 of their household contacts, and their pet (canine). Additionally, MLST was performed on 12 *E. coli* isolates, and the results showed the presence of ST131 and ST394 (2 isolates each), and ST398, ST4977, ST8420, ST349, ST101, ST58, ST457, and ST6011 (1 isolate each).

**Figure 4.**
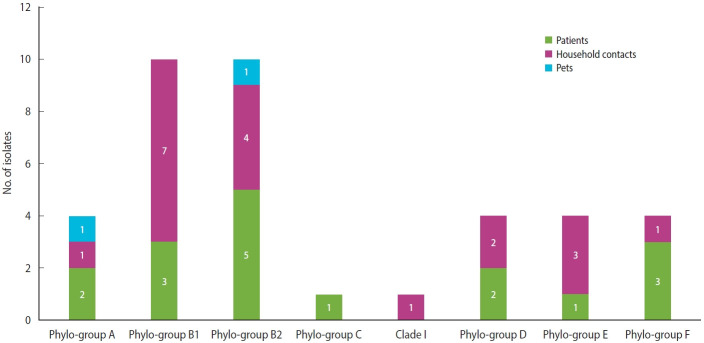
Phylogroup distribution of Escherichia coli. E. coli isolates colonizing hemodialysis patients and their household contacts.

Regarding the classification by the Clermont scheme, 38 isolates of *E. coli* were analyzed, where the main phylogroups identified were B1 and B2, each with 26.3% (n= 10), followed by phylogroups A, D, E, and F, each with 10.5% (n= 4). Finally, phylogroup C and clade 1 were identified, each with 2.6% (n= 1) (Figure 4).

### Factors associated with colonization

The potential factors associated with colonization were analyzed for ESBL-producing Gram-negative bacilli, and the results are presented in Tables 2 and 3, for hemodialysis patients and household contacts, respectively. Colonization was positively associated with not washing hands (PR, 1.89; 95% CI, 1.38 to 2.58) and the presence of comorbidities, such as cancer (PR, 2.57; 95% CI, 1.42 to 4.63). However, washing hands after using the toilet (PR, 0.46; 95% CI, 0.30 to 0.71) acted as a protective factor. Other factors such as previous antibiotic consumption did not show associations with colonization by ESBL-producing Gram-negative bacilli.

## DISCUSSION

The results of this study demonstrated a high frequency of colonization not only in hemodialysis patients, but also in their household contacts. Although several studies have evaluated colonization by BR-GNB, this work started from a high-risk group and took into account a population that constantly circulates between hospital and the community. This allowed us to refine our understanding of the behavior of colonization between community and hospital environments and to observe the role of homes as a possible reservoir and disseminator of this type of bacteria.

The frequency of BR-GNB colonization in hemodialysis patients was similar to that reported in a previous study carried out in hemodialysis patients from the same renal unit, where 41.2% and 11.5% of patients were colonized by ESBL-producing and carbapenem-resistant Gram-negative, respectively [4]. Our results exceed the prevalence of BR-GNB reported in other studies, such as Wendt et al. [18], of a kidney unit in Germany, where colonization by multi-resistant Gram-negative bacteria was 10.4%. This difference could be explained by the high hygiene standards and the strict antibiotic use policies in that institution.

Regarding the household contacts of hemodialysis patients, there are few studies evaluating colonization by BR-GNB in this population. However, the frequency reported in this study exceeds the values reported for the general community, which are between 9.8% and 14% according to some studies [19-21]. In studies carried out in relatives of other types of patients, such as patients with hemolytic uremic syndrome, a frequency of colonization by *E. coli* of 12% was reported [22], while Adler et al. [23] found ESBL-producing enterobacteria in 9.1% of relatives of patients hospitalized in rehabilitation centers. One of the reasons for these differences may be that the household contacts of hemodialysis patients are in constant transit between the hospital and the community due to their bond of care with the patients, which favors contact with the hospital environment. In addition, the frequencies of colonization are also affected by geographic location, and Colombia is considered an endemic country for ESBL-producing Gram-negative bacilli [24]. Nonetheless, the consumption of antibiotics in the household contacts was very high, which may favor the selection pressure for resistant bacteria in this population.

The importance of colonization by BR-GNB in the general community lies in the fact that these people can be the main source of transport and dissemination of these microorganisms, so characterizing this population men it possible to know the behavior of resistance at the local level [25]. Accordingly, the relatives of hemodialysis patients can transport these resistant bacteria in the community and in the hospital; this contact with health institutions by accompanying patients has been described as a possible cause that strengthens the community transmission of ESBL-producing *Enterobacteriaceae* [20]. However, the percentage of colonization by BR-GNB in animals is similar to the data reported in a study in New Zealand, where the 6.4% of dogs and cats were colonized by ESBL-producing *E. coli*, which indicates that companion animals can also be reservoirs and acquire resistant bacteria in homes or veterinary clinics [26].

The presence of resistant bacteria in the community makes this environment an important source of acquisition of these microorganisms, not only in homes but also in places where there is frequent contact with other people or with potentially contaminated surfaces, such as supermarkets, public transport, and schools. In this sense, it is worth highlighting the importance of designing and implementing educational strategies that improve knowledge about bacterial resistance and the proper use of antibiotics in the general community, based on the particular needs and epidemiological behavior of resistance in different populations.

The PFGE and MLST results show that the high diversity of isolates in our population and confirm *E. coli* as a microorganism with high genetic plasticity. Likewise, it suggests a strong selection pressure in different places that behave as sources of colonization, which can occur at the hospital level or from community spaces, since our population is in constant transit between the 2 environments. Once colonized, patients and their household contacts become carriers of this type of bacteria and can help it to spread.

However, the genetic relationship found in isolates from different members in 3 families confirms that bacteria can be shared in homes between inhabitants, including animals, and shows homes as potential reservoirs for bacterial resistance. This has been described in other populations, which place the relationship between colonizing strains of ESBL-producing *E. coli* between members of the same household between 8% and 27%. An exchange of bacteria has also been seen between domestic animals and their caregivers, with a relationship reported in between 25% and 27% of cases [27,28]. This highlights the importance of taking into account homes, household contacts, and companion animals in strategies to control the spread of resistant bacteria.

Concerning *E. coli*, classification by the Clermont system made it possible to differentiate commensal strains from intestinal and extraintestinal pathogenic variants; this is important to stablish a relationship between colonizing strains and disease, which is information of vital importance in this type of patient [29,30]. In this study, the most frequent *E. coli* phylogroups were B1, which has been identified as a cause of infections in dialysis-dependent patients [31], and B2, which has been considered the main phylogroup worldwide in causing extraintestinal disease [32] and, in turn, has been documented as one of the main colonizers of the gastrointestinal tract of patients with other chronic diseases [33,34], being related to ST131, which is characterized by presenting a resistance profile to beta-lactam antibiotics [35]. Likewise, the isolates from pets in our study were classified into phylogroups A and B2, coinciding with previous reports, where dogs were considered potential transmitters of pathogenic *E. coli* to their caregivers [36].

Regarding the factors associated with colonization, good hygiene practices have been documented as protective factors for colonization by resistant bacteria, which is consistent with the data obtained in this study, where the absence of handwashing was related to colonization [37,38]. Similarly, having chronic diseases such as cancer and using antibiotics are among the most frequently described risk factors for colonization by ESBL-GNB [39].

Finally, regarding the resistance mechanisms detected, most of the isolates were E. coli carriers of ESBL, especially CTX-M-G1. This agrees with what has been reported in previous studies of hemodialysis patients in Colombia, where CTX-M-G1 was present in 47.9% of the *E. coli* isolates evaluated [40]. In general, beta-lactamases of the CTX-M type are widely distributed worldwide and are endemic in Latin America, North America, and Asia [41], so it is common to find microorganisms with these enzymes colonizing and infecting different population groups throughout the world [42-45].

Regarding to carbapenem-resistant Gram-negative bacilli, other studies have reported from 2.4% to 4.2% of isolates carrying KPC in patients with chronic kidney disease. The difference from our study may be because this work is cross-sectional, but colonization can act intermittently, which could have influenced the results [45,46]. Nonetheless, carbapenemases were not detected in *P. aeruginosa* isolates; however, their resistance may be due to a combination of intrinsic resistance with modifications in the expression of porins or expulsion pumps. It should be noted that various reports point to an increase in BR-GNB, especially carriers of ESBL in the community, which shows the importance of continuing with studies aimed at knowing the real magnitude of colonization in this environment and the factors associated with it to establish more effective intervention strategies.

In conclusion, the results of this study showed that there was a high frequency of colonization by BR-GNB both in hemodialysis patients and in their household contacts, confirming the presence of this type of bacteria both in people with contact with the hospital environment and in the community. Likewise, the most frequently isolated microorganism was ESBL-producing *E. coli*, and it was confirmed that the strains circulating in our population have pathogenic potential since most belonged to the B1 and B2 phylogroups. The diversity observed also suggests that, within the community, there are different sources from which these bacteria can be acquired, which implies that surveillance of bacterial resistance should be strengthened at the community level, and that preventative measures should be implemented. As for limitations and perspectives, this population could not be followed, which could improve the understanding of the behavior and duration of colonization by BR-GNB. Likewise, studies involving whole-genome sequencing could clarify the directionality of transmission between patients and household contacts, which would help direct more assertive control strategies.

## Figures and Tables

**Table 1.** Clinical and epidemiological characteristics of hemodialysis patients and their household contacts, according to their colonization status by BR-GNB

Characteristics		All subjects		Patients			Household contacts		
		Colonization	Non-colonization	Colonization	Non-colonization	p-value	Colonization	Non- colonization	p-value
Total		41 (32.5)	85 (67.5)	21 (58.3)	15 (41.7)	-	20 (22.2)	70 (77.8)	-
Women		24 (58.5)	56 (65.9)	10 (47.6)	6 (40.0)	0.765	14 (70.0)	50 (71.4)	0.402
Age, median (IQR) (yr)		55 (26-66)	48 (27-64)	58 (39-72)	64 (50-71)	0.392	29 (14-59)	40 (18-59)	0.291
Traveling in the last year		10 (24.4)	24 (28.2)	3 (14.3)	3 (20.0)	>0.999	7 (35.0)	21 (30.0)	0.239
Medical history (last year)									
	Medicine	10 (24.4)	11 (12.9)	7 (33.3)	5 (33.3)	0.813	3 (15.0)	6 (8.6)	0.659
	Hospitalization	15 (36.6)	12 (14.1)	13 (61.9)	5 (33.3)	0.095	2 (10.0)	7 (10.0)	>0.999
	Surgery	14 (34.1)	16 (18.8)	12 (57.1)	6 (40.0)	0.317	2 (10.0)	10 (14.3)	0.685
	Antibiotic consumption	20 (48.8)	25 (29.4)	13 (61.9)	5 (33.3)	0.019	7 (35.0)	20 (28.6)	>0.999
Comorbidities									
	Diabetes	13 (31.7)	10 (11.8)	9 (42.9)	3 (20.0)	0.059	4 (20.0)	7 (10.0)	0.405
	Heart disease	7 (17.1)	6 (7.1)	7 (33.3)	4 (26.7)	0.387	0 (0.0)	2 (2.9)	>0.999
	Dyslipidemia	6 (14.6)	9 (10.6)	6 (28.6)	3 (20.0)	0.451	0 (0.0)	6 (8.6)	0.586
	Arterial hypertension	20 (48.8)	24 (28.2)	18 (85.7)	11 (73.3)	0.684	2 (10.0)	13 (18.6)	>0.999
Hand-washing habits									
	Before eating	28 (68.3)	66 (77.6)	17 (81.0)	12 (80.0)	>0.999	11 (55.0)	54 (77.1)	0.365
	Before toilet	18 (43.9)	36 (42.4)	7 (33.3)	7 (46.7)	0.102	11 (55.0)	29 (41.4)	0.031
	After toilet	37 (90.2)	82 (96.5)	19 (90.5)	15 (100)	0.487	18 (90.0)	67 (95.7)	0.215
	After getting home	29 (70.7)	60 (70.6)	14 (66.7)	7 (46.7)	0.535	15 (75.0)	53 (75.7)	>0.999
Gastrointestinal symptoms (last 6 mo)									
	Vomit	8 (19.5)	7 (8.2)	4 (19.0)	1 (6.7)	>0.999	4 (20.0)	6 (8.6)	0.073
	Diarrhea	16 (39.0)	23 (27.1)	9 (42.9)	4 (26.7)	0.137	7 (35.0)	19 (27.1)	0.543
	Other	2 (4.9)	5 (5.9)	1 (4.8)	0 (0.0)	>0.999	1 (5.0)	5 (7.1)	0.586
Feeding habits									
	Pork consumption	37 (90.2)	72 (84.7)	19 (90.5)	11 (73.3)	0.391	18 (90.0)	61 (87.1)	0.681
	Chicken consumption	29 (70.7)	73 (85.9)	15 (71.4)	11 (73.3)	0.717	14 (70.0)	62 (88.6)	0.120
	Beef consumption	36 (87.8)	73 (85.9)	16 (76.2)	12 (80.0)	>0.999	20 (100)	61 (87.1)	0.353
	Tap-water consumption	25 (61.0)	68 (80.0)	10 (47.6)	1 (93.3)	0.059	15 (75.0)	54 (77.1)	>0.999

Values are presented as number (%). BR-GNB, beta-lactam-resistant Gram-negative bacilli.

**Table 2.** Bivariate and multivariate analyses to identify potential factors associated with colonization by ESBL-producing Gram-negative bacilli in hemodialysis patients

Variable		Bivariate analysis	p-value	Multivariate analysis^1^	p-value
Market in the neighborhood store		0.71 (0.33, 1.53)	0.386	0.65 (0.31, 1.37)	0.262
Preparation of food by the mother		2.31 (1.52, 3.49)	<0.001	2.79 (1.30, 5.97)	0.008
Travel last year		0.94 (0.39, 2.26)	0.886	0.89 (0.39, 1.99)	0.769
Consumption of foods of animal origin					
	Pork	1.70 (0.52, 5.59)	0.382	1.66 (0.49, 5.57)	0.414
	Beef	1.07 (0.49, 2.35)	0.863	1.04 (0.46, 2.33)	0.925
	Chicken	0.83 (0.44, 1.59)	0.579	0.79 (0.40, 1.56)	0.499
Tap water consumption		0.56 (0.31, 1.00)	0.048	0.57 (0.32, 1.02)	0.059
	Handwashing				
	Before eating	0.91 (0.43, 1.90)	0.792	1.01 (0.49, 2.08)	0.988
	After toilet	0.50 (0.36, 0.70)	<0.001	0.46 (0.30, 0.71)	<0.001
	Not washing hands	1.94 (1.40, 2.70)	<0.001	1.89 (1.38, 2.58)	<0.001
History in last year					
	Home medicine	0.92 (0.47, 1.83)	0.819	1.00 (0.48, 2.06)	0.990
	Hospitalization	1.71 (0.87, 3.36)	0.117	1.64 (0.83, 3.23)	0.154
	Surgery	1.38 (0.72, 2.61)	0.332	1.44 (0.77, 2.71)	0.252
	Antibiotic consumption	2.17 (1.05, 4.47)	0.036	2.13 (1.04, 4.36)	0.038
	Third-generation cephalosporins or fluoroquinolones	1.16 (0.52, 2.60)	0.714	1.10 (0.48, 2.50)	0.816
Comorbidities:					
	Cancer	1.94 (1.40, 2.70)	<0.001	2.57 (1.42, 4.63)	0.002
	Diabetes mellitus	1.80 (1.00, 3.23)	0.048	2.13 (1.17, 3.85)	0.013

Values are presented as prevalence ratio (95% confidence interval). ESBL, extended-spectrum beta-lactamase.

1 Adjusted by age.

**Table 3.** Bivariate and multivariate analyses to identify potential factors associated with colonization by ESBL-producing Gram-negative bacilli in household contacts

Variables	Bivariate analysis	p-value	Multivariate analysis^1^	p-value
Market in the neighborhood store	4.79 (1.36, 16.83)	0.015	4.54 (1.33, 15.53)	0.016
Preparation of food by the mother	1.94 (0.62, 6.11)	0.256	2.25 (0.73, 6.96)	0.158
Travel last year	0.53 (0.14, 1.98)	0.345	0.55 (0.15, 1.96)	0.357
Consumption of foods of animal origin				
Pork	1.42 (0.35, 5.71)	0.624	1.35 (0.33, 5.50)	0.680
Chicken	0.42 (0.21, 0.82)	0.011	0.43 (0.23, 0.82)	0.011
Tap water consumption	0.83 (0.39, 1.77)	0.627	0.80 (0.37, 1.72)	0.570
Handwashing				
Before eating	0.74 (0.33, 1.66)	0.467	0.76 (0.35, 1.65)	0.494
After toilet	0.66 (0.15, 2.81)	0.570	0.63 (0.14, 2.85)	0.546
History in the last year				
Home medicine	0.95 (0.24, 3.72)	0.945	1.00 (0.29, 3.50)	0.994
Hospitalization	0.46 (0.08, 2.58)	0.379	0.48 (0.13, 1.79)	0.275
Surgery	0.43 (0.12, 1.50)	0.185	0.48 (0.14, 1.60)	0.232
Antibiotic consumption	0.86 (0.34, 2.19)	0.752	0.93 (0.37, 2.33)	0.874
Comorbidities				
Diabetes mellitus	1.87 (0.69, 5.11)	0.221	2.68 (0.91, 7.89)	0.073
Sharing towels with the hemodialysis patient	1.94 (0.94, 4.01)	0.072	1.85 (0.91, 3.75)	0.088
Sharing soap with the hemodialysis patient	1.28 (0.68, 2.39)	0.441	1.36 (0.71, 2.59)	0.352
Sharing a bathroom with the hemodialysis patient	1.89 (0.69, 5.15)	0.214	1.87 (0.66, 5.25)	0.237
Sharing meals with the hemodialysis patient	1.21 (0.87, 1.68)	0.261	1.21 (0.88, 1.67)	0.235
Cleaning up the hemodialysis patient	0.55 (0.24, 1.30)	0.175	0.61 (0.26, 1.41)	0.248

Values are presented as prevalence ratio (95% confidence interval). ESBL, extended-spectrum beta-lactamase.

1 Adjusted by age.

## SUPPLEMENTARY MATERIALS

Supplementary Material 1. Flow chart illustrating the inclusion of participants.
