# Supplementary material for: High frequency of colonization by extended-spectrum beta-lactamase-producing Gram-negative bacilli in hemodialysis patients and their household contacts in Colombia: dissemination between the community and the hospital
Source: Epidemiol Health. 2022 Aug 27;44:e2022069. doi: 10.4178/epih.e2022069 (PMC9943636; doi:10.4178/epih.e2022069)
Supplement: Supplementary Material 1. — Flow chart illustrating the inclusion of participants. [file epih-44-e2022069-Supplementary-1.pptx]

## Slide 1
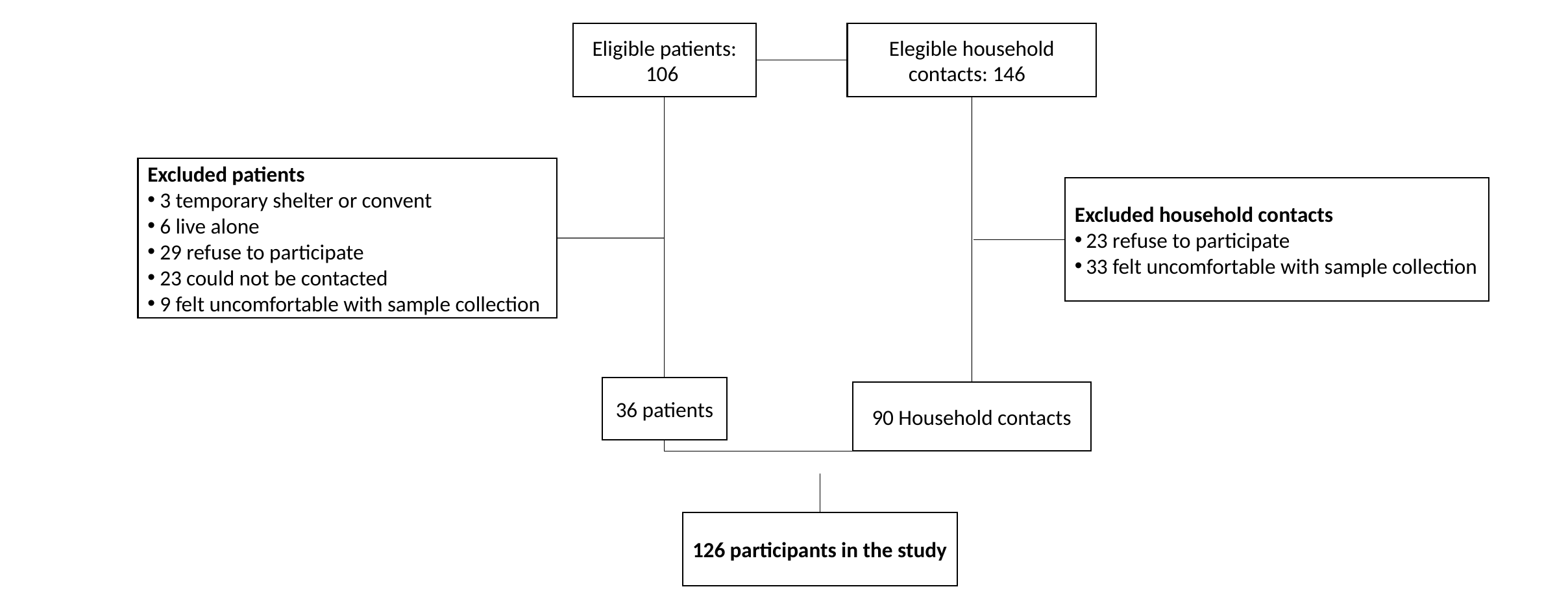

Eligible patients: 106
Elegible household contacts: 146
Excluded patients
 3 temporary shelter or convent
 6 live alone
 29 refuse to participate
 23 could not be contacted
 9 felt uncomfortable with sample collection
Excluded household contacts
23 refuse to participate
33 felt uncomfortable with sample collection
36 patients
90 Household contacts
126 participants in the study
